# “Tuberculosis in advanced HIV infection is associated with increased expression of IFNγ and its downstream targets”

**DOI:** 10.1186/s12879-018-3127-4

**Published:** 2018-05-15

**Authors:** Sheetal Verma, Peicheng Du, Damalie Nakanjako, Sabine Hermans, Jessica Briggs, Lydia Nakiyingi, Jerrold J. Ellner, Yukari C. Manabe, Padmini Salgame

**Affiliations:** 1 Department of Medicine, Center for Emerging Pathogens, Rutgers University New Jersey Medical School, Newark, NJ USA; 2 Office of Advanced Research Computing, Rutgers University New Jersey Medical School, Newark, NJ USA; 3 Infectious Diseases Institute, Makerere University College of Health Sciences, Kampala, Uganda; 4 Amsterdam Institute of Global Health and Development, Amsterdam Medical Center, Amsterdam, Netherlands; 5 Department of Medicine, Johns Hopkins University School of Medicine, Baltimore, MD USA; 6 Present address: UCSF, Division of Infectious Diseases, San Francisco, CA USA; 7 Department of Medicine, Boston Medical Center and Boston University School of Medicine, Boston, MA USA

**Keywords:** Tuberculosis, HIV, Biomarker, TLR, Inflammation, IRIS, Transcriptomics, Cytokines, CXCL10, IFNγ, *FcGR1A*, *BATF2*

## Abstract

**Background:**

Tuberculosis (TB) is the major cause of death in Human Immunodeficiency Virus (HIV)-infected individuals. However, diagnosis of TB in HIV remains challenging particularly when HIV infection is advanced. Several gene signatures and serum protein biomarkers have been identified that distinguish active TB from latent infection. Our study was designed to assess if gene expression signatures and cytokine levels would distinguish active TB in advanced HIV.

**Methods:**

We conducted a case-control study of whole blood RNA-Seq and plasma cytokine/chemokine analysis in HIV-infected with CD4^+^ T cell count of ≤ 100 cells/μl, with and without active TB. Next, the overlap of the differentially expressed genes (DEG) with the published signatures was performed and then receiver operator characteristic (ROC) analysis was done on small gene discriminators to determine their performance in distinguishing TB in advanced HIV. ELISA was performed on plasma to evaluate cytokine and chemokine levels.

**Results:**

Hierarchical clustering of the transcriptional profiles showed that, in general, HIV-infected individuals with TB (TB-HIV) clustered separately from those without TB. IPA indicated that the TB-HIV signature was characterized by an increase in inflammatory signaling pathways. Analysis of overlaps between DEG in our data set with published TB signatures revealed that significant overlap was seen with one TB signature and one TB-IRIS signature. ROC analysis revealed that transcript levels of *FcGR1A* (AUC = 0.85) and *BATF2* (AUC = 0.82), previously reported as consistent single gene classifiers of active TB irrespective of HIV status, performed successfully even in advanced HIV. Plasma protein levels of IFNγ, a stimulator of *FcGR1A* and *BATF2*, and CXCL10, also up-regulated by IFNγ, accurately classified active TB (AUC = 0.98 and 0.91, respectively) in advanced HIV. Neither of these genes nor proteins distinguished between TB and TB-IRIS.

**Conclusions:**

Gene expression of *FcGR1A* and *BATF2*, and plasma protein levels of IFNγ and CXCL10 have the potential to independently detect TB in advanced HIV. However, since other lung diseases were not included in this study, these final candidates need to be validated as specific to TB in the advanced HIV population with TB.

**Electronic supplementary material:**

The online version of this article (10.1186/s12879-018-3127-4) contains supplementary material, which is available to authorized users.

## Background

Tuberculosis (TB) is the leading cause of death in HIV-infected persons. The majority (74%) of TB-HIV coinfected persons reside in Sub-Saharan Africa [1]. Overall, HIV-infected are 20–30 times more likely to develop active TB than those without HIV infection, with an annual risk as high as 5–15% per year [2, 3]. The transition from latent TB infection (LTBI) to active disease in HIV-infected depends on host immunity and on the level of immunosuppression [4, 5]. The CD4^+^ T cell count is only an approximate measure of the degree of immunosuppression at the population level and thus an inaccurate marker of the likelihood of reactivation in a HIV-infected host [6–8].

Along the spectrum of infection, individuals with asymptomatic active disease (subclinical TB) [9] are clinically unrecognized and often progress to active symptomatic TB disease [10]. In a high prevalence setting, 8.5% of asymptomatic, HIV-infected persons had positive sputum cultures for *Mycobacterium tuberculosis* (Mtb) [11]. Even when sputum culture is used to exclude active TB, asymptomatic cases may be misclassified particularly in the immunosuppressed where disseminated disease may predominate [12]. HIV-infected with occult TB are at risk of unmasking TB-IRIS, which has significant morbidity and mortality [7, 13]. Therefore, new approaches to the diagnosis of TB in this group are imperative.

Transcriptional profiling studies have identified several different biosignatures that can differentiate active TB from LTBI [14–20] and from other related inflammatory diseases [16, 17, 21]. Two of these, the 27-gene signature identified in the study by Kaforou et al., [16], and the 251-gene signature reported by Dawany et al., [22], performed well even in HIV-infected individuals. The biosignatures derived from these multiple cohorts do not show significant overlap. However, publically available databases provide a wealth of material for meta-analysis and derivation of a biomarker signature that can potentially function across cohorts. Using this approach, a targeted Real Time-PCR (RT–PCR) array was designed by Maertzdorf and colleagues [23] based on two of their microarray datasets [24, 25] and then applied to a new cohort from India. This strategy generated a 4-biomarker set (*GBP1*, *IFITM3*, *P2RY14* and *ID3*) that performed satisfactorily even in HIV-coinfected individuals. Subsequently, a 3-gene set consisting of *GBP5*, *CD64* and *GZMA* was identified that accurately separated TB from OPD (other pulmonary diseases) [26]. Other studies have shown that *CD64*, *FcGR1B* and *LTF* are differentially expressed in TB versus other lung diseases [17, 27, 28]. Another meta-analysis [29], using a total of 14 datasets identified a different set of three genes (*GBP5*, *DUSP3*, and *KLF2*) that were highly diagnostic for active tuberculosis in HIV infected and uninfected populations. Validation studies confirmed that this 3-gene set separated active tuberculosis from healthy controls, latent tuberculosis and other diseases. The usefulness of this gene set in monitoring treatment response is under-scored by the observation that the expression of the 3-gene set declined during treatment of patients with active tuberculosis [29]. In another multi-site study [30], a targeted approach was used to validate genes identified in single-site populations. This study found that *FcGR1A* was the most reliable classifier of active TB and its expression was not confounded by HIV status or ethnic background. The performance of *FcGR1A* was not compromised even in advanced HIV [30]. Similarly, another recent study analyzed new and previously published data on blood transcriptional profiles and found that elevated transcript levels of *BATF2* can also robustly discriminate active TB from healthy controls [31]. However, the study found elevated *BATF2* levels even in the absence of TB in HIV negative individuals who presented with various other infectious diseases. The authors concluded that *BATF2* transcript may have better value as a test for ruling out TB. We therefore tested the accuracy of *FcGR1A,* which was not confounded by HIV status, and *BATF2*, which had better negative predictive value for TB, in discriminating active TB in the context of coinfection with HIV.

Biomarkers for tuberculosis have also been identified through serum proteomic profiles. A comparison of TB cases to healthy controls identified fibrinogen degradation product (FDP) [32] and Orosomucoid (ORM) as potential biomarkers [33]. Another study found that levels of serum amyloid A, transthyretin, C-reactive protein, and neopterin discriminated patients with TB from other respiratory disorders (ORD) and inflammatory diseases [34]. In comparison to LTBI and ORD, serum samples from TB patients expressed a biomarker panel of 8 proteins [35]. This study also found that in the HIV coinfected TB patients, the composition of the host protein biomarker panel was slightly different and consisted of 10 host proteins [35]. Other metabolomic profiling studies have also provided additional biomarker panels that discriminate TB patients from healthy controls [36–38]. In a recent study using Somascans, an aptamer-based proteomic platform, De Groote and colleagues measured over 4000 host proteins and identified a 6-marker signature for active TB that included SYWC, kallistatin, C9, gelsolin, testican-2, and aldolase C [39].

Thus, the aim of this study was to determine if gene expression signature and plasma protein biomarkers would distinguish active TB in severely immunosuppressed TB-HIV participants prior to initiation of TB treatment or anti-retroviral therapy (ART).

## Methods

### Study design and participant recruitment

Inpatients from Mulago National Tertiary Referral Hospital and outpatients from the Infectious Diseases Institute at Makerere University College of Health Sciences with serologically confirmed HIV-1 infection, who were ≥ 18 years of age with a CD4^+^ T cell count ≤ 100 cells/μL, were recruited to a prospective cohort study between March and December 2013. The participants were included on the basis of a CD4^+^ T cell count documented in the chart. CD4^+^ T cell counts were rechecked at baseline for 2 participants, and were found to be slightly above 100 cells/μl. They were still included in the study. A targeted medical history as well as socio-demographic information was collected. Patients were systematically screened for TB at the Infectious Diseases Institute. HIV status was gathered either from the patient’s chart or was retested, if unknown. During follow-up, the one participant who was HIV positive, on ART and developed TB was reclassified as such. None of the included patients had any other documented opportunistic infection. Participants were assigned to one of 3 groups: new diagnosis of smear-positive or microbiologically confirmed TB and ART-naïve; smear or microbiologically-confirmed TB within 180 days of initiating ART (unmasked TB); no signs or symptoms of TB and ART-naïve. Participants were excluded if they had received more than 2 days of anti-TB medication within the previous 60 days. Blood samples were only collected at baseline. Participants were only followed to determine 6 month outcome and to verify that they did not have unmasking TB or immune reconstitution inflammatory syndrome (IRIS).

### Blood sample collection and processing

Whole blood was collected into PAXgene Blood RNA tubes (supplied by Qiagen, catalog # 762125) and plasma was frozen at − 80 °C at the time of enrollment. Whole blood was also collected into Becton Dickinson vacutainer acid citrate dextrose (ACD) tubes for collection of peripheral blood mononuclear cells (PBMC). PAXgene Blood RNA tubes were stored at − 80 °C and PBMC were stored in liquid nitrogen until further use.

### RNA sequencing (RNA-Seq) and data analysis

RNA was isolated from whole blood using the PAXgene Blood RNA Kit (Qiagen, catalog # 762164). Total RNA input was amplified using MessageAmp™ II aRNA Amplification Kit (ThermoFisher Scientific). 100 ng of amplified RNA was used to prepare the library. Following standard instructions for fragmentation, purification, 3′ and 5′ adapter ligation and reverse transcription, PCR amplicons were purified using AMPure XP beads and the library was quantified. This library was used for RNA Sequencing on Illumina Hiseq2500. On average 49–50 million basepair (bp) reads were produced per sample. Quality check of reads was performed with the software FastQC. Low quality reads and adapters were trimmed with Cutadapt [40]. Trimmed reads were mapped to the human genome GRCh38/hg19 with STAR [41], and the expression level for each gene was counted with HTSeq [42] according to gene annotations from Ensembl. The Bioconductor DESeq package in R [43] was used to normalize the counts and call differential expressions. Principal Component Analysis (PCA) was used for data visualization. Hierarchical clustering was performed with the gplots package in R. The ROC curve was plotted with the pROC package in R. Functional and network analyses of differentially expressed genes were performed using Ingenuity Pathway Analysis (IPA, Ingenuity Systems, https://www.qiagenbioinformatics.com/products/ingenuity-pathway-analysis/, Redwood City, CA).

### Multiplex immunoassays

Pre-coated 10-spot MULTI-SPOT® plates with capture antibodies were purchased [catalog # K15054D-2; Meso Scale Discovery (MSD), Rockville, MD, USA]. The assays are based on the principle of electrochemiluminescence (ECL) sandwich ELISA. Briefly, plasma samples were centrifuged for 20 min at 2000 g and diluted 2-fold for cytokine analytes and 4-fold for chemokine targets prior to using. Calibrator controls, detection antibody mix and read buffer were prepared as per manufacturer’s instructions. The prepared samples and calibrators were added to the plates and incubated overnight at 2–8 °C. Plates were then washed and incubated with detection antibody solution at room temperature with shaking for 2 h. After washing, 2X Read Buffer was added to each well and plates were analyzed on MESO Quickplex SQ120. The calculations to establish calibration curves and determine analyte concentrations were carried out using the MSD DISCOVERY WORKBENCH® analysis software. Protein levels of IFN-γ, IL-6, IL-8, TNF-α, Eotaxin, MCP-1, MCP-4, MIP-1α, MIP-1β, CCL17, IL-12/IL-23p40, IL-15, IL-16, IL-17A, VEGF and CXCL10 were assessed. The software generated calibration curves by fitting the signals from the calibrators to a 4-parameter logistic model with a 1/Y2 weighting. Analyte concentrations were determined from the ECL signals by back-fitting to the calibration curves.

### Statistical analysis

Descriptive statistics were used to characterize the study population. Patient baseline characteristics were compared by Fischer’s exact for categorical data and Wilcoxon rank sum test for continuous variables. Non-parametric analysis of plasma cytokine levels was done using Mann Whitney U-test.

## Results

### Participant demographics and baseline characteristics

Sixteen HIV-infected participants with smear (+) or microbiologically confirmed TB (TB-HIV), 15 HIV-infected participants (HIV) that had no clinical symptoms of TB and 2 TB-HIV participants undergoing treatment with ART (unmasked TB) were studied. Table 1 shows the demographic characteristics and outcomes for 32 participants, except for one TB-HIV for whom data was not available. Most of the study subjects had a CD4^+^ T cell count ≤ 100 cells/μl. Greater than 90% of participants with HIV alone were reported alive after 6 months from their initial admission, but almost 60% participants with TB-HIV diagnosis did not survive; 5 of the 16 TB-HIV died within 16 days of enrollment. None of the HIV-infected (no TB) had been treated with ART prior to study. Blood samples were collected prior to start of TB treatment in all participants, except one patient who had received 2 days of TB treatment.

**Table 1:** Demographic Characteristics and Participant Outcomes

	Total^a^	TB-HIV	HIV	*p*-value
	(*n* = 32)	(*n* = 17)	(*n* = 15)	
Female	62% (20/32)	47% (8/17)	80% (12/15)	0.08^
Median Age (range)	32 [18–53]	30 [18–52]	36 [24–53]	0.43^^
Initial CD4^+^ (cells/μL)	50 [4–105]^b^	23 [4–105]	67 [11–96]	0.15^^
Time to ART start (days)	14 [6–80]^c^	14.5 [12–80]	12 [6–51]	0.08^^
IRIS	20% (5/25)	45% (5/11)	0% (0/14)	0.13^
Alive at 6 months	75% (24/32)	59% (10/17)	93% (14/15)	0.03^

^a^Demographic data available for 32 study participants. Whole blood RNA-Seq analysis done for 33 participants

^b^The initial CD4 count for 2 participants was 103 and 105 cells/μl; however when repeated for the study, the counts were less than 100 cells/μl

^c^19 observations

^p-value calculated by Fischer’s exact

^^p-value calculated by Wilcoxon rank sum test

Age given is median age, range is entire range of ages. CD4^+^ range is entire range of CD4^+^ T cell counts

### Distinct blood transcriptional profile distinguishes TB and HIV coinfected from HIV alone

**Fig. 1:**
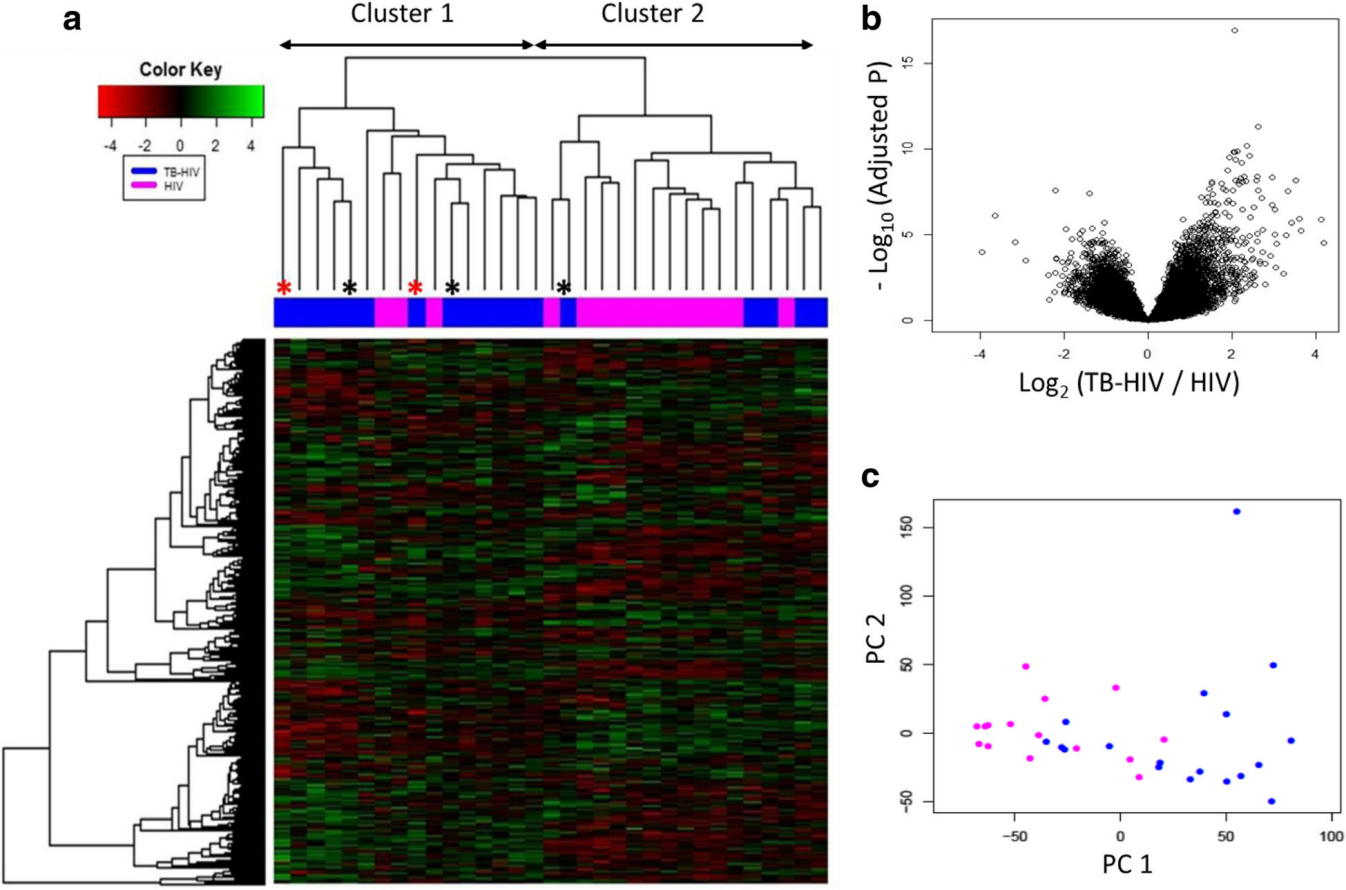
Global expression analysis of RNA-Seq data shows two main clusters. Heatmap of top 10,000 differentially expressed genes from RNA-Seq data. RNA-Seq was performed on whole blood samples from 16 TB-HIV, 15 HIV and 2 TB-HIV on ART. Dendrograms represent unsupervised hierarchical clustering of samples. Expression scale is log2 of normalized counts. TopHat and Cufflinks algorithmic tools were used for comprehensive expression analysis of high-throughput RNA-Seq data. Red * indicates the 2 TB-HIV subjects on ART that developed IRIS and black * indicates other subjects with TB-HIV IRIS (**a**). Differential gene expression seen in the Volcano plot represents participants with TB-HIV (including 2 TB-HIV on ART) vs. HIV only, from the RNA-Seq data. The x-axis is log2 of the fold change of TB-HIV vs. HIV only. The y-axis is log odds of the FDR adjusted *p* value (**b**). PCA plot of PC1 vs. PC2 of RNA-Seq data of TB-HIV including those that received ART (Blue) and HIV only (Magenta) (**c**)

**Fig. 2:**
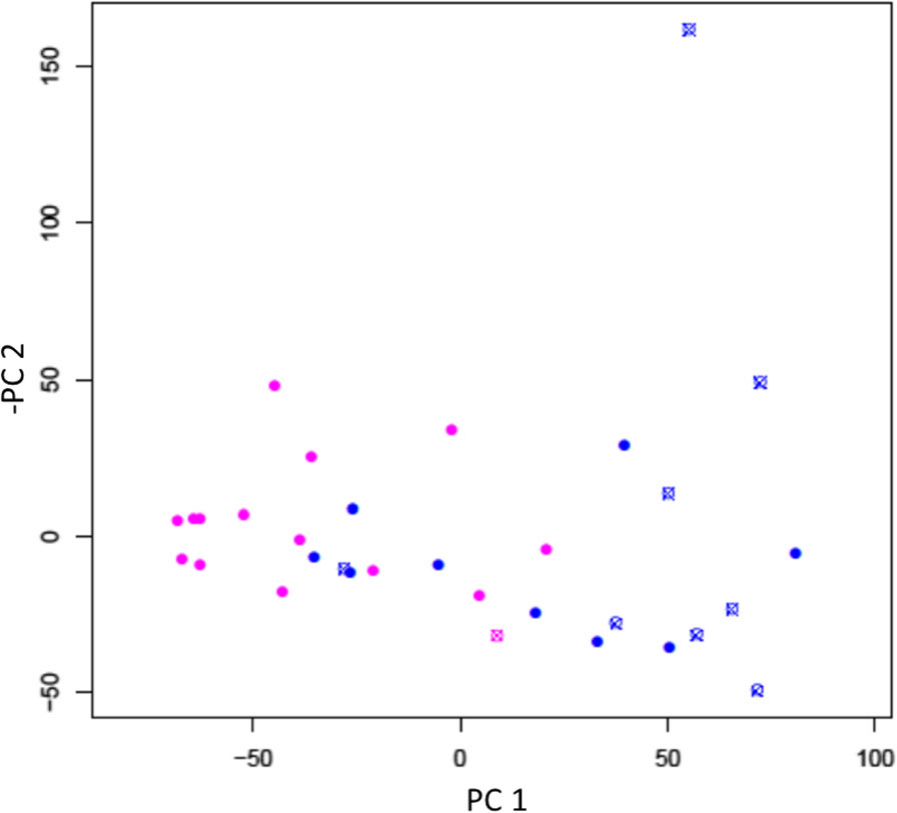
Absence of mortality-related clustering by PCA. Plot of PC1 vs. PC2 of RNA-Seq data shows participants with TB-HIV including those that received ART (Blue) and HIV only (Magenta). Blue solid circles are TB-HIV subjects that were alive after six months of enrollment, whereas blue crossed circles show TB-HIV participants that succumbed to infection. Magenta solid circles and crossed circles are representative of HIV subjects alive and not alive after six months, respectively

Whole blood RNA-Seq analysis was performed on all 33 participants. Hierarchical clustering of the transcriptional profiles based on logarithmic plotting of normalized counts showed two clustering patterns that were non-random, that is, within cluster variance looks smaller than between cluster variance (Fig. 1a). The top 50 DEG are listed in Additional file 1: Figure S1. Differential expression of genes between TB-HIV and HIV is reported in the volcano plot (Fig. 1b). Eleven of the 16 HIV participants with active TB (TB-HIV) clustered separately (Cluster 1) from HIV-infected participants (HIV) that were asymptomatic, with no clinical or microbiological evidence of TB (Cluster 2). The remaining 5 TB-HIV segregated with Cluster 2 and the 3 HIV segregated with Cluster 1. The 2 TB-HIV being treated with ART also segregated with Cluster 1 (Fig. 1a). The data were further analyzed using PCA. Consistent with the heat map, plot of PC1 vs. PC2 also corroborated that the participants segregated into two main groups (Fig. 1c). The 11 TB-HIV and 3 HIV that belonged to Cluster 1 in the global expression analysis also segregated as one group by PCA and included the 2 TB-HIV that had previously received treatment with ART and then developed unmasked TB. Additionally, both clustering and PCA resulted in the same 5 TB-HIV patients separating out from the majority grouping of TB-HIV patients (Fig. 1c). Although some participants with TB-HIV did not survive, PCA and RNA-Seq patterns seen previously were not indicative of a mortality-related clustering (Fig. 2). Of note, the only subject that died in the HIV group was in fact one of the 3 HIV that segregated with Cluster 1.

### Data mining using IPA reveals enrichment of innate inflammatory pathway genes in TB-HIV coinfected participants

Network, function, and pathway analyses were generated using IPA (Qiagen). Differential expression gene list comparing TB-HIV samples to HIV only, with *p*-value < 0.01 and a log fold change greater than 2, was generated and uploaded to IPA. Analysis showed that the top five canonical pathways contributing to over-expression of genes related to the following signaling pathways: Role of Macrophages, Fibroblasts and Endothelial Cells in Rheumatoid Arthritis; IL-10, p38 MAPK and TLR signaling, Hepatic Fibrosis/Hepatic Stellate Cell Activation (Table 2). Predicted upstream regulators of this signature were IL-1, Immunoglobulin, PGR, lipopolysaccharide, CSF2 (Table 2). The top five canonical pathways contributing to down-regulated expression of genes related to EIF2, Primary Immunodeficiency, B cell development, Granzyme A and Regulation of eIF4 and p70S6K signaling pathways (Table 2). Predicted upstream regulators of these pathways were IL-15, MYCN, Alefacept (a genetically engineered anti-inflammatory drug), T Cell Receptor and lipopolysaccharide (Table 2).

**Table 2:** IPA analysis enlisting top five canonical pathways and upstream regulators in TB-HIV and HIV gene sets

Top pathways (upregulated)	*p*-value	Overlap	Overlapping Genes
Role of Macrophages, Fibroblasts and Endothelial Cells in Rheumatoid Arthritis	6.01E-06	6.3% (18/287)	SOCS3,ICAM1,TLR8,CEBPD, IRAK3,IL1R1, CREB5, PDGFC, FCGR1A, IL18R1, IL18RAP, IL1R2,FOS, NFKBIA,MAPK14,TLR5,TLR1,TCF7L2
IL-10 Signaling	3.24E-05	11.8% (8/68)	IL1R2,FOS, SOCS3, IL4R,NFKBIA, MAPK14,IL1R, IL18RAP
p38 MAPK Signaling	5.71E-05	8.5% (10/117)	IL1R2, MAPK14,TIFA,DUSP1, MKNK1, IRAK3,IL1R1, CREB5, IL18RAP, HSPB1
Toll-like Receptor Signaling	3.68E-04	9.6% (7/73)	FOS,NFKBIA,MAPK1,TLR5, TLR1,TLR8, IRAK3
Hepatic Fibrosis / Hepatic Stellate Cell Activation	5.09E-04	6.1% (11/181)	IL1R2,IL4R, ICAM1,HGF, FGFR1,IGF1R, COL4A2,IL1R, IFNAR2, PDGFC, IL18RAP
Upstream Regulators (upregulated)	*p*-value of overlap		
Immunoglobulin	1.51E-13		
PGR	3.23E-08		
Lipopolysaccharide	4.31E-08		
CSF2	4.81E-07		
IL-1	4.98E-07		
EIF2 Signaling	3.40E-08	7.5% (13/173)	RPS7,RPS6, RPL4, RPLP1, RPL23A, RPL22L1, RPL37,EIF4A2, RPL23,RPS17, RPS4,RPL13A,RPS11
Primary Immunodeficiency Signaling	6.06E-06	13.6% (6/44)	RFX5,LCK, ZAP70, CD8A,UNG, IGHD
B Cell Development	1.67E-04	14.8% (4/27)	SPN,CD79B, HLA-DMB,IGHD
Granzyme A Signaling	6.82E-04	17.6% (3/17)	PRF1,H1FX, APEX1
Regulation of eIF4 and p70S6K Signaling	7.64E-04	4.9% (7/143)	RPS7,RPS6, EIF4A2, RPS17,RPS4X, RPS11,ITGA4
Upstream Regulators (downregulated)	*p*-value of overlap		
MYCN	2.72E-09		
Alefacept	5.84E-08		
TCR	2.59E-06		
Lipopolysaccharide	2.89E-06		
IL-15	9.25E-06		

### Overlap of differentially expressed genes in TB-HIV versus HIV with existing TB signatures

A 27 [16] and a 251-gene [22] signature have been reported to accurately segregate TB-HIV coinfected from HIV only. We therefore tested whether the 749 differentially expressed genes in our dataset overlapped significantly with published gene signatures. As shown in Table 3, there was significant overlap with the 27-gene signature, however significant overlap was not observed with the 251-gene signature. IPA pointed to an enrichment of innate inflammatory pathway genes in TB-HIV coinfected participants in our cohort, similar to that reported by Lai et al. [44]. Here, a 43-transcript signature characterized by innate Toll-like receptor and inflammasome signaling predicts the development of immune reconstitution inflammatory syndrome (IRIS) in TB-HIV [44]. We found that the DEG that were over-expressed in Cluster 1 had significant overlap with this 43-transcript signature (Table 3). We also found significant overlap with the 16-gene blood transcriptional signature that predicts risk of progression to TB disease [45].

**Table 3:** Overlap of DEGs with existing gene signatures

Gene list source	Genes in Signature	Overlap with DE genes (Current study)	Common	Overlap *p*-value
Kaforou et al. (PMID: 24167453)	27	CD79B, DUSP3, FAM20A, FLVCR2, FCGR1A, ANKRD22	6	0.004707
Dawany et al. (PMID: 24587128)	251	ZNF516, SMAD7, ITGA4, FLVCR2, MSRB2, C9orf91, IFNAR2, PPBP, ARL4C, GK HLA-DMB	11	0.6178
Lai et al. (PMID: 26399326)	43	MAPK14, SIPA1L2, CDK5RAP2, ANXA3, BCL2A1, DOK3, ACSL1, TPST1, PFKFB3, BASP1, GPR97, TLR5	12	5.39E-08
Zak et al. (PMID: 27017310)	16	FCGR1A, ANKRD22, BATF2	3	0.0001189

### Cytokine levels in plasma

**Fig. 3:**
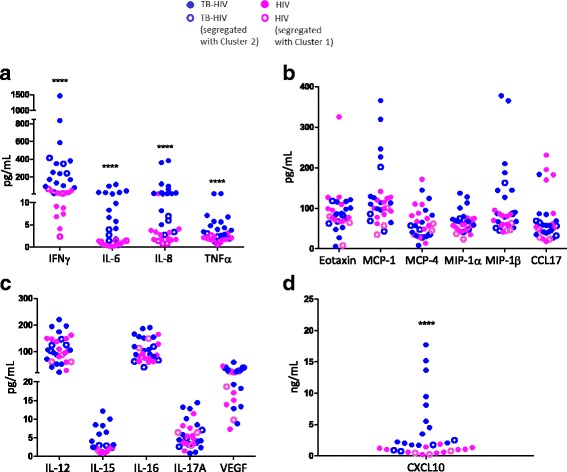
Increased levels of inflammatory mediators in plasma of TB-HIV. Plasma samples from 31 participants were analyzed for cytokines and chemokines using a multi-analyte detection system (Meso Scale Discovery, Rockville, MD, USA). Plasma samples from 1 individual from each of the TB-HIV and HIV were not available. Data is represented as absolute value for each participant for a given cytokine or chemokine (**a**-**d**). Significance was determined by Mann Whitney U test. ****p < 0.0001

IPA predicted up-regulated expression of innate inflammatory genes in TB-HIV. We therefore quantified the levels of 16 pro-inflammatory mediators in the plasma of TB-HIV and HIV to determine if a cytokine/chemokine signature would distinguish the two groups. Significantly higher levels of IFNγ and CXCL10 were seen in TB-HIV compared to HIV (*p* < 0.0001) (Fig. 3a and d). IL-6, IL-8 and TNF were also significantly up-regulated in the TB-HIV (p < 0.0001) (Fig. 3a). Levels of key leukocyte chemotactic agents MIP-1α and MIP-1β were up-regulated as well in TB-HIV, compared to those with HIV only (Fig. 3b). Interestingly, the 3 HIV-infected participants whose transcriptional profile was different from the others with HIV alone exhibited pro-inflammatory cytokine levels equivalent to the other HIV-infected patients (Fig. 3). Also noteworthy were the 5 TB-HIV participants that clustered away from the parent TB-HIV Cluster 1 in RNA-Seq, yet showed cytokine levels comparable to their parent group (Fig. 3). Together, these data indicate that: i) soluble markers of immune activation are elevated in TB-HIV compared to HIV and ii) the TB-HIV and HIV who did not segregate with their fellow members in their transcriptional profile, nonetheless had cytokine profiles similar to their fellow members. Although there were no biases due to gender, age or other evaluated parameters, it was not clear why some samples were misclassified in RNA-Seq.

### *FcGR1A* and *BATF2* transcript levels, and IFNγ and CXCL10 plasma protein levels singly classify active TB

A successful diagnostic test requires minimal number of markers, and consequently several studies have pursued this goal to come up with 3–10 genes as sufficient for successfully classifying active TB from controls [20, 23, 29]. As shown in Table 4, we obtained AUC scores for these signatures to determine how well they would perform in discriminating TB in advanced HIV. The AUC score for the 3-gene transcriptional signature from the study by Sweeney et al. [29] was 0.89. The 4-gene signature from the Maertzdorf study [23] and the 10-gene signature from the Sambarey study [20] performed well, with AUC scores of 0.91 and 0.92, respectively (Table 4).

**Table 4:** Receiver Operator Characteristic AUC scores of different signatures to discriminate active TB from controls

Gene list source	HIV	Genes in signature	AUC	95% CI
Sweeney et al. (PMID: 26907218)	yes	DUSP3, GBP5, KLF2	0.89	0.771–1
Laux de Costa et al. (PMID: 26025597)	no	GBP5, FcGR1A, GZMA	0.87	0.7575–1
Maertzdorf et al. (PMID: 26682570)	yes	GBP1, IFITM3, P2RY14, ID3	0.91	0.8001–1
Sambarey et al. (PMID: 28065665)	no	FcGR1A, HK3, RAB13, RBBP8, IFI44L, TIMM10, BCL6, SMARCD3, CYP4F3, SLPI	0.92	0.8453–1

**Fig. 4:**
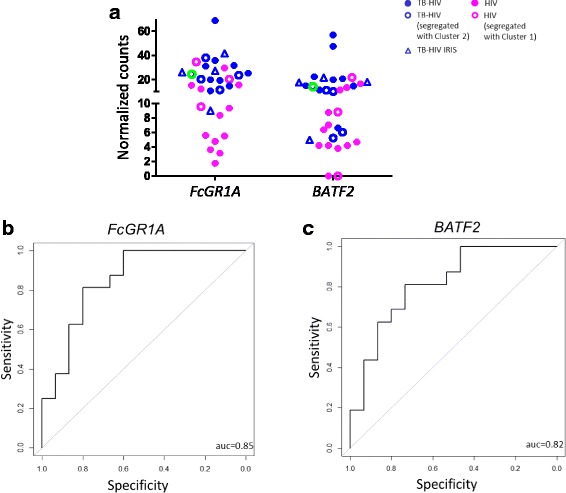
Classification of active TB in advanced HIV using *FcGR1A* and *BATF2*. Normalized counts obtained from RNA-Seq analysis for all 33 participants are plotted for *FcGR1A* and *BATF2* (**a**). Green open circle is representative of sample from one TB-HIV participant, who segregated with Cluster 2 and also developed IRIS (**a**). ROC AUC score for each target was obtained using the pROC package in R.Functional (**b** and **c**)

*FcGR1A* is part of the discriminatory gene set in a number of biomarker studies [14, 15, 46] and as a single marker it fairly discriminates active TB from latent infection regardless of HIV status or genetic background [30]. Given that *FcGR1A* is differentially expressed in our study between TB-HIV and HIV (Fig. 4a), we performed ROC analysis to determine if *FcGR1A* was also a good classifier of active TB in advanced HIV. We found that *FcGR1A* correctly classified 85% of the patients [AUC = 0.85; 95% CI: 0.7095–0.9905] (Fig. 4b). A recent report found *BATF2* transcript was also an accurate classifier of active TB in both HIV-uninfected and infected individuals [31]. In our advanced HIV cohort, *BATF2* transcript levels discriminated active TB with an AUC score of 0.82 [95% CI: 0.6642–0.9692] (Fig. 4a and c).

**Fig. 5:**
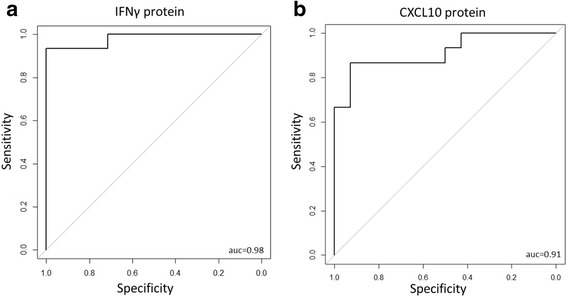
Classification of active TB in advanced HIV using IFNγ and CXCL10. Protein concentrations from patient plasma samples were used to determine ROC AUC scores for IFNγ and CXCL10 (**a** and **b**). AUC scores were obtained using the pROC package in R.Functional

IFNγ is a potent stimulator of *FcGR1A* and *BATF2* expression and its protein levels are significantly upregulated in TB-HIV (Fig. 3). We therefore obtained ROC AUC of 0.98 for the diagnostic potential of IFNγ protein expression in our dataset [95% CI: 0.9402–1] (Fig. 5a). Next, we also performed similar ROC analysis to determine the performance of CXCL10 since its plasma levels were upregulated in TB-HIV (Fig. 3) and its expression is also induced by IFNγ. CXCL10 accurately classified TB with an AUC score of 0.91 [95% CI: 0.8078–1] (Fig. 5b).

### Development of IRIS

The cohort was followed for 6 months. Five of the TB-HIV coinfected (including the two that were undergoing treatment with ART at enrollment) developed IRIS (Table 1). The participants who developed IRIS segregated with Cluster 1 and had comparable levels of IFNγ and CXCL10 with respect to the TB-HIV cases that did not develop IRIS (Additional file 2: Figure S2). These findings indicate that in our cohort, participants with TB showed a high innate inflammatory signature irrespective of whether they developed IRIS or not.

## Discussion

The significant outcome of this study is the finding that differential transcript levels of *FcGR1A* and *BATF2* and protein expression levels of IFNγ and CXCL10 robustly classified active TB in advanced HIV, including the 3 HIV-infected and 5 TB-HIV individuals whose gene expression pattern was not consistent with their fellow members. *FcGR1A* is expressed on most myeloid cells [47] and it activates a number of effector functions, including phagocytosis, antigen-presentation, the production of cytokines and reactive oxygen species and antibody-mediated cellular cytotoxicity (reviewed in [48]). The study by Sutherland and colleagues [30] found that *FcGR1A* could correctly segregate 75% of the HIV participants into active TB disease or not. In our study, *FcGR1A* performed better by correctly classifying 85% of the advanced HIV participants. Treatment of TB results in a significant reduction of *FcGR1A* expression [49] suggesting that monitoring *FcGR1A* may provide a useful tool to monitor treatment response in advanced HIV.

Basic leucine zipper transcription factor ATF-like (*BATF*) *2*, is a transcription factor that belongs to the activator protein 1 family of transcription factors and contains the basic leucine zipper domain. *BATF2* mediates pro-inflammatory responses by associating with Interferon regulatory factor 1 (IRF1). Of note, *BATF2* gene expression is induced by type I IFNs [50] and by LPS, IFNγ and Mtb in macrophages [51]. Based on data from multiple study cohorts, Roe and colleagues proposed the application of *BATF2* as a robust discriminator of active TB from healthy individuals [31]. Their study also demonstrated that the classification accuracy was not compromised by HIV infection. Performance of *BATF2* in our cohort (AUC 0.82) was equivalent to that reported by Roe et al. (AUC 0.84).

Expression of *FcGR1A* is induced by IFNγ [52] and consistent with this we found IFNγ levels to accurately segregate TB-HIV patients from HIV. In the seminal study of O’Garra and colleagues [14], an IFN signature was associated with TB. However, it was surprising to see a strong IFNγ response in patients with CD4^+^ counts < 100. Other innate cell types, including γδ T cells and NK cells secrete IFNγ during TB infection (reviewed in [53]). It would be interesting to investigate the cell type(s) producing IFNγ in advanced HIV since it may provide opportunities for developing flow cytometry-based diagnostic tools. Even if the specificity of such an assay is low, it would serve as a potential point-of-care triage test. CXCL10 is selectively up-regulated in TB pleural effusions compared with malignant effusions [54–56] and in lateral flow based test it showed promise as a diagnostic tool for pleural TB [57]. CXCL10 is also one of the seven-marker serum protein biosignature for the diagnosis of active TB disease in African primary healthcare clinic attendees with signs and symptoms suggestive of TB [58]. In our study plasma levels of CXCL10 accurately classified TB in 91% of the TB-HIV patients, indicating the potential of this analyte as a diagnostic biomarker.

A study from Lai and colleagues found a consistent over-abundance of 43 genes encompassing innate immune mediators of the TLR and inflammasome signaling pathways in TB-IRIS participants, 2 weeks before they developed IRIS when compared to non-IRIS controls [44]. It is not clear why the TB-HIV participants in our cohort who did not progress to IRIS also had a high innate inflammatory signature. One possibility is the timing of the blood sample collection for transcriptomics. The blood samples in our study were obtained from participants prior to anti-TB treatment, whereas in the Lai study [44] they were collected following approximately 1 month of anti-TB therapy. Given that even 2 weeks of anti-TB treatment is sufficient to significantly down-modulate the TB gene signature [59], the baseline samples collected prior to ART, but post anti-TB treatment, from TB-HIV coinfected participants may have a lower expression of inflammatory genes. Therefore in the Lai study, using these baseline samples as comparator, the increase in inflammatory genes associated with TLR and inflammasome signaling could be detected as early as 0.5 week following ART in only those progressing to IRIS. The mechanisms leading to enhanced inflammation in untreated TB versus in those developing IRIS may be different, but since the mediators of inflammation are non-specific, the gene signature for TB in the HIV-infected and that for IRIS may overlap. Thus, our findings would suggest that use of gene transcript sets as biomarkers for predicting IRIS should take into account the TB treatment status of the TB-HIV subjects.

Although the sample size in this study is small, nonetheless, pathway analysis (through IPA) of the transcriptional data provides a few clues to the underlying mechanisms leading to the enhanced inflammatory response in the TB-HIV group. For example, TLR signaling, p38 MAPK signaling and role of macrophages, fibroblasts and endothelial cells in Rheumatoid Arthritis were some of the top upregulated pathways. Interleukin-1β, one of the upregulated upstream regulators is clearly essential for anti-mycobacterial immunity but excessive amounts of the cytokine is pro-inflammatory and can cause tissue damage (reviewed in [60]). Granulocyte-macrophage colony-stimulating factor (GM-CSF), also referred to as colony-stimulating factor 2 (CSF2), another upregulated upstream regulator, also contributes to anti-mycobacterial immunity [61]. However, in the context of autoimmune disease, GM-CSF is a strong inducer of tissue inflammation and plays a critical role in disease progression (reviewed in [62]). EIF2 signaling and primary immunodeficiency signaling were the top downregulated pathways. Phosphorylation of Eukaryotic initiation factor 2 (eIF2) is activated by diverse stress responses and results in repression of global translation, except for the translation of select genes, including *ATF4* (activating transcription factor 4). ATF4 is a master regulator of the integrated stress response [63, 64]. Interestingly, treating macrophages with the drug Guanabenz increased the phosphorylation level of eIF2α and resulted in elevated mRNA levels of IL-6 and GM-CSF [65]. This leads to the conjecture that the decreased EIF2 signaling may be related to the enhanced inflammatory response seen in TB-HIV. Granzyme A signaling was another downregulated pathway. Since Granzyme-A is expressed by NK cells and CD8^+^ cytotoxic T cells and is a tryptase that induces a caspase-independent cell death [68], its downregulation is suggestive of functional defects in the two immune cell types. Alefacept, is a dimeric fusion protein made up of the extracellular CD2-binding portion of the human leukocyte function–associated antigen 3 (LFA-3) linked to the Fc portion of human IgG1 [66]. Alefacept preferentially targets CD4^+^ and CD8^+^ effector memory T cells [66] and therefore in the context of HIV fits as one of the upstream regulators that was found to be downregulated. IL-15, a cytokine critical for NK cell proliferation and memory CD8^+^ T cell development, was another upstream regulator that was downregulated [67]. Overall, the IPA data point to upregulated and downregulated pathways as well as regulators that could together result in increased inflammation in TB-HIV. This is further reflected in the increased levels of inflammatory biomarkers in plasma.

## Conclusion

Overall, we found that plasma protein levels of IFNγ and CXCL10 and gene expression of *FcGR1A* and *BATF2* have the potential of independently detecting TB in advanced HIV. However, a limitation of this study is the small sample size and lack of inclusion of other relevant “conditions” in HIV-infected. Future endeavors should include other inflammatory and infectious diseases. Additional focus should be given to longitudinal cohort studies to determine whether measure of FcGR1A, *BATF2*, IFNγ and CXCL10 could be developed into an accurate and rapid diagnostic test for subclinical TB disease in advanced HIV, particularly in countries where both infections are endemic.

## Additional files

Additional file 1: **Figure S1.** A heatmap of top 50 genes differentially expressed between TB-HIV and HIV. Heat map showing differential gene expression of top 50 genes using largest value of adjusted fold change and *p* = 0.01 in TB-HIV as compared to HIV-only group. The x-axis is log2 of the fold change between the two groups. The y-axis is log odds of the FDR adjusted *p* value. (PPTX 297 kb)

Additional file 2: **Figure S2.** Progression to IRIS does not alter plasma cytokine/chemokine levels in TB-HIV participants. Plasma samples obtained from TB-HIV and TB-HIV with IRIS were analyzed for cytokines and chemokines using the Meso Scale Discovery 30-plex multi-analyte detection system. Data is represented as absolute value for each participant for a given cytokine or chemokine. Significance was determined by Mann Whitney U test. (PPTX 121 kb)

## Abbreviations

ACD: Acid citrate dextrose
ART: Anti-retroviral therapy
AUC: Area under the curve
BP: Basepair
CI: Confidence intervals
DEG: Differentially expressed genes
HIV: Human Immunodeficiency Virus
IFN: Interferon
IPA: Ingenuity pathway analysis
LPS: Lipopolysaccharide
LTBI: Latent TB infection
Mtb: *Mycobacterium tuberculosis*
OPD: Other pulmonary diseases
ORD: Other respiratory disorders
PBMC: Peripheral blood mononuclear cells
PCA: Principal component analysis
ROC: Receiver operator characteristic
TB: Tuberculosis
TB-IRIS: TB-immune reconstitution inflammatory syndrome

## References

[1] WHO (2015). World Health Organization: global tuberculosis report 2015.

[2] Corbett EL, Watt CJ, Walker N, Maher D, Williams BG, Raviglione MC, Dye C (2003). The growing burden of tuberculosis: global trends and interactions with the HIV epidemic. Arch Internal Med.

[3] Nunn P, Williams B, Floyd K, Dye C, Elzinga G, Raviglione M (2005). Tuberculosis control in the era of HIV. Nat Rev Immunol.

[4] Daley CL, Small PM, Schecter GF, Schoolnik GK, McAdam RA, Jacobs WR, Hopewell PC (1992). An outbreak of tuberculosis with accelerated progression among persons infected with the human immunodeficiency virus. An analysis using restriction-fragment-length polymorphisms. N Engl J Med.

[5] Alland D, Kalkut GE, Moss AR, McAdam RA, Hahn JA, Bosworth W, Drucker E, Bloom BR (1994). Transmission of tuberculosis in new York City. An analysis by DNA fingerprinting and conventional epidemiologic methods. N Engl J Med.

[6] Salgame P, Geadas C, Collins L, Jones-Lopez E, Ellner JJ (2015). Latent tuberculosis infection--revisiting and revising concepts. Tuberculosis (Edinb).

[7] Manabe YC, Breen R, Perti T, Girardi E, Sterling TR (2009). Unmasked tuberculosis and tuberculosis immune reconstitution inflammatory disease: a disease spectrum after initiation of antiretroviral therapy. J Infect Dis.

[8] Lawn SD, Wood R, Wilkinson RJ. Changing concepts of "latent tuberculosis infection" in patients living with HIV infection. Clin Dev Immunol. 2011; p. 9, Article ID 980594. 10.1155/2011/980594. 10.1155/2011/980594 PMC2948911 20936108

[9] Mtei L, Matee M, Herfort O, Bakari M, Horsburgh CR, Waddell R, Cole BF, Vuola JM, Tvaroha S, Kreiswirth B (2005). High rates of clinical and subclinical tuberculosis among HIV-infected ambulatory subjects in Tanzania. Clin Infect Dis.

[10] Corbett EL, Bandason T, Cheung YB, Munyati S, Godfrey-Faussett P, Hayes R, Churchyard G, Butterworth A, Mason P (2007). Epidemiology of tuberculosis in a high HIV prevalence population provided with enhanced diagnosis of symptomatic disease. PLoS Med.

[11] Oni T, Burke R, Tsekela R, Bangani N, Seldon R, Gideon HP, Wood K, Wilkinson KA, Ottenhoff TH, Wilkinson RJ (2011). High prevalence of subclinical tuberculosis in HIV-1-infected persons without advanced immunodeficiency: implications for TB screening. Thorax.

[12] Nakiyingi L, Moodley VM, Manabe YC, Nicol MP, Holshouser M, Armstrong DT, Zemanay W, Sikhondze W, Mbabazi O, Nonyane BA (2014). Diagnostic accuracy of a rapid urine lipoarabinomannan test for tuberculosis in HIV-infected adults. J Acquir Immune Defic Syndr.

[13] Lawn SD, Harries AD, Meintjes G, Getahun H, Havlir DV, Wood R (2012). Reducing deaths from tuberculosis in antiretroviral treatment programmes in sub-Saharan Africa. AIDS.

[14] Berry MP, Graham CM, McNab FW, Xu Z, Bloch SA, Oni T, Wilkinson KA, Banchereau R, Skinner J, Wilkinson RJ (2010). An interferon-inducible neutrophil-driven blood transcriptional signature in human tuberculosis. Nature.

[15] Jacobsen M, Repsilber D, Gutschmidt A, Neher A, Feldmann K, Mollenkopf HJ, Ziegler A, Kaufmann SH (2007). Candidate biomarkers for discrimination between infection and disease caused by Mycobacterium tuberculosis. J Mol Med (Berl).

[16] Kaforou M, Wright VJ, Oni T, French N, Anderson ST, Bangani N, Banwell CM, Brent AJ, Crampin AC, Dockrell HM (2013). Detection of tuberculosis in HIV-infected and -uninfected African adults using whole blood RNA expression signatures: a case-control study. PLoS Med.

[17] Bloom CI, Graham CM, Berry MP, Rozakeas F, Redford PS, Wang Y, Xu Z, Wilkinson KA, Wilkinson RJ, Kendrick Y (2013). Transcriptional blood signatures distinguish pulmonary tuberculosis, pulmonary sarcoidosis, pneumonias and lung cancers. PLoS One.

[18] Ottenhoff TH, Dass RH, Yang N, Zhang MM, Wong HE, Sahiratmadja E, Khor CC, Alisjahbana B, van Crevel R, Marzuki S (2012). Genome-wide expression profiling identifies type 1 interferon response pathways in active tuberculosis. PLoS One.

[19] Lu C, Wu J, Wang H, Wang S, Diao N, Wang F, Gao Y, Chen J, Shao L, Weng X (2011). Novel biomarkers distinguishing active tuberculosis from latent infection identified by gene expression profile of peripheral blood mononuclear cells. PLoS One.

[20] Sambarey A, Devaprasad A, Mohan A, Ahmed A, Nayak S, Swaminathan S, D'Souza G, Jesuraj A, Dhar C, Babu S (2017). Unbiased identification of blood-based biomarkers for pulmonary tuberculosis by modeling and mining molecular interaction networks. EBioMed.

[21] Maertzdorf J, Weiner J, Mollenkopf HJ, Bauer T, Prasse A, Muller-Quernheim J, Kaufmann SH (2012). Common patterns and disease-related signatures in tuberculosis and sarcoidosis. Proc Nat Acad Sci USA.

[22] Dawany N, Showe LC, Kossenkov AV, Chang C, Ive P, Conradie F, Stevens W, Sanne I, Azzoni L, Montaner LJ (2014). Identification of a 251 gene expression signature that can accurately detect M. Tuberculosis in patients with and without HIV co-infection. PLoS One.

[23] Maertzdorf J, McEwen G, Weiner J, Tian S, Lader E, Schriek U, Mayanja-Kizza H, Ota M, Kenneth J, Kaufmann SH (2016). Concise gene signature for point-of-care classification of tuberculosis. EMBO Mol Med.

[24] Maertzdorf J, Repsilber D, Parida SK, Stanley K, Roberts T, Black G, Walzl G, Kaufmann SH (2011). Human gene expression profiles of susceptibility and resistance in tuberculosis. Genes Immun.

[25] Maertzdorf J, Ota M, Repsilber D, Mollenkopf HJ, Weiner J, Hill PC, Kaufmann SH (2011). Functional correlations of pathogenesis-driven gene expression signatures in tuberculosis. PLoS One.

[26] Laux da Costa L, Delcroix M, Dalla Costa ER, Prestes IV, Milano M, Francis SS, Unis G, Silva DR, Riley LW, Rossetti ML (2015). A real-time PCR signature to discriminate between tuberculosis and other pulmonary diseases. Tuberculosis (Edinb).

[27] Joosten SA, Fletcher HA, Ottenhoff TH (2013). A helicopter perspective on TB biomarkers: pathway and process based analysis of gene expression data provides new insight into TB pathogenesis. PLoS One.

[28] Maertzdorf J, Weiner J, Mollenkopf HJ, Network TB, Bauer T, Prasse A, Muller-Quernheim J, Kaufmann SH (2012). Common patterns and disease-related signatures in tuberculosis and sarcoidosis. Proc Nat Acad Sci USA.

[29] Sweeney TE, Braviak L, Tato CM, Khatri P (2016). Genome-wide expression for diagnosis of pulmonary tuberculosis: a multicohort analysis. Lancet Respir Med.

[30] Sutherland JS, Loxton AG, Haks MC, Kassa D, Ambrose L, Lee JS, Ran L, van Baarle D, Maertzdorf J, Howe R (2014). Differential gene expression of activating Fcgamma receptor classifies active tuberculosis regardless of human immunodeficiency virus status or ethnicity. Clin Microbiol Infect.

[31] Roe JK, Thomas N, Gil E, Best K, Tsaliki E, Morris-Jones S, Stafford S, Simpson N, Witt KD, Chain B (2016). Blood transcriptomic diagnosis of pulmonary and extrapulmonary tuberculosis. JCI Insight.

[32] Liu J, Jiang T, Wei L, Yang X, Wang C, Zhang X, Xu D, Chen Z, Yang F, Li JC (2013). The discovery and identification of a candidate proteomic biomarker of active tuberculosis. BMC Infect Dis.

[33] Zhang J, Wu X, Shi L, Liang Y, Xie Z, Yang Y, Li Z, Liu C, Yang F (2012). Diagnostic serum proteomic analysis in patients with active tuberculosis. Clin Chim Acta.

[34] Agranoff D, Fernandez-Reyes D, Papadopoulos MC, Rojas SA, Herbster M, Loosemore A, Tarelli E, Sheldon J, Schwenk A, Pollok R (2006). Identification of diagnostic markers for tuberculosis by proteomic fingerprinting of serum. Lancet.

[35] Achkar JM, Cortes L, Croteau P, Yanofsky C, Mentinova M, Rajotte I, Schirm M, Zhou Y, Junqueira-Kipnis AP, Kasprowicz VO (2015). Host protein biomarkers identify active tuberculosis in HIV uninfected and co-infected individuals. EBioMed.

[36] Weiner J, Parida SK, Maertzdorf J, Black GF, Repsilber D, Telaar A, Mohney RP, Arndt-Sullivan C, Ganoza CA, Fae KC (2012). Biomarkers of inflammation, immunosuppression and stress with active disease are revealed by metabolomic profiling of tuberculosis patients. PLoS One.

[37] Mahapatra S, Hess AM, Johnson JL, Eisenach KD, DeGroote MA, Gitta P, Joloba ML, Kaplan G, Walzl G, Boom WH (2014). A metabolic biosignature of early response to anti-tuberculosis treatment. BMC Infect Dis.

[38] Frediani JK, Jones DP, Tukvadze N, Uppal K, Sanikidze E, Kipiani M, Tran VT, Hebbar G, Walker DI, Kempker RR (2014). Plasma metabolomics in human pulmonary tuberculosis disease: a pilot study. PLoS One.

[39] De Groote MA, Sterling DG, Hraha T, Russell TM, Green LS, Wall K, Kraemer S, Ostroff R, Janjic N, Ochsner UA (2017). Discovery and validation of a six-marker serum protein signature for the diagnosis of active pulmonary tuberculosis. J Clin Microbiol.

[40] Martin M. Cutadapt removes adapter sequences from high-throughput sequencing reads. EMBnet.journal. 2011;17(1):10–12. 10.14806/ej.17.1.200.

[41] Dobin A, Davis CA, Schlesinger F, Drenkow J, Zaleski C, Jha S, Batut P, Chaisson M, Gingeras TR (2013). STAR: ultrafast universal RNA-seq aligner. Bioinformatics.

[42] Anders S, Pyl PT, Huber W (2015). HTSeq--a Python framework to work with high-throughput sequencing data. Bioinformatics.

[43] Anders S, Huber W (2010). Differential expression analysis for sequence count data. Gen Biol.

[44] Lai RP, Meintjes G, Wilkinson KA, Graham CM, Marais S, Van der Plas H, Deffur A, Schutz C, Bloom C, Munagala I (2015). HIV-tuberculosis-associated immune reconstitution inflammatory syndrome is characterized by toll-like receptor and inflammasome signalling. Nat Commun.

[45] Zak DE, Penn-Nicholson A, Scriba TJ, Thompson E, Suliman S, Amon LM, Mahomed H, Erasmus M, Whatney W, Hussey GD (2016). A blood RNA signature for tuberculosis disease risk: a prospective cohort study. Lancet.

[46] Kassa D, Ran L, Jager W, van den Broek T, Jacobi R, Mekonen M, Messele T, Haks MC, Ottenhoff TH, van Baarle D (2016). Discriminative expression of whole blood genes in HIV patients with latent and active TB in Ethiopia. Tuberculosis (Edinb).

[47] Bournazos S, Wang TT, Ravetch JV (2016). The role and function of Fcgamma receptors on myeloid cells. Microbiol Spectr.

[48] van der Poel CE, Spaapen RM, van de Winkel JG, Leusen JH (2011). Functional characteristics of the high affinity IgG receptor, FcgammaRI. J Immunol.

[49] Cliff JM, Lee JS, Constantinou N, Cho JE, Clark TG, Ronacher K, King EC, Lukey PT, Duncan K, Van Helden PD (2013). Distinct phases of blood gene expression pattern through tuberculosis treatment reflect modulation of the humoral immune response. J Infect Dis.

[50] Su ZZ, Lee SG, Emdad L, Lebdeva IV, Gupta P, Valerie K, Sarkar D, Fisher PB (2008). Cloning and characterization of SARI (suppressor of AP-1, regulated by IFN). Proc Nat Acad Sci USA.

[51] Roy S, Guler R, Parihar SP, Schmeier S, Kaczkowski B, Nishimura H, Shin JW, Negishi Y, Ozturk M, Hurdayal R (2015). Batf2/Irf1 induces inflammatory responses in classically activated macrophages, lipopolysaccharides, and mycobacterial infection. J Immunol.

[52] Pearse RN, Feinman R, Ravetch JV (1991). Characterization of the promoter of the human gene encoding the high-affinity IgG receptor: transcriptional induction by gamma-interferon is mediated through common DNA response elements. Proc Nat Acad Sci USA.

[53] Bhatt K, Verma S, Ellner JJ, Salgame P (2015). Quest for correlates of protection against tuberculosis. Clin Vaccine Immunol.

[54] Pokkali S, Das SD, R L (2008). Expression of CXC and CC type of chemokines and its receptors in tuberculous and non-tuberculous effusions. Cytokine.

[55] Okamoto M, Kawabe T, Iwasaki Y, Hara T, Hashimoto N, Imaizumi K, Hasegawa Y, Shimokata K (2005). Evaluation of interferon-gamma, interferon-gamma-inducing cytokines, and interferon-gamma-inducible chemokines in tuberculous pleural effusions. J Lab Clin Med.

[56] Dheda K, Van-Zyl Smit RN, Sechi LA, Badri M, Meldau R, Symons G, Khalfey H, Carr I, Maredza A, Dawson R (2009). Clinical diagnostic utility of IP-10 and LAM antigen levels for the diagnosis of tuberculous pleural effusions in a high burden setting. PLoS One.

[57] Sutherland JS, Mendy J, Gindeh A, Walzl G, Togun T, Owolabi O, Donkor S, Ota MO, Kon Fat ET, Ottenhoff TH (2016). Use of lateral flow assays to determine IP-10 and CCL4 levels in pleural effusions and whole blood for TB diagnosis. Tuberculosis (Edinb).

[58] Chegou NN, Sutherland JS, Malherbe S, Crampin AC, Corstjens PL, Geluk A, Mayanja-Kizza H, Loxton AG, van der Spuy G, Stanley K (2016). Diagnostic performance of a seven-marker serum protein biosignature for the diagnosis of active TB disease in African primary healthcare clinic attendees with signs and symptoms suggestive of TB. Thorax.

[59] Bloom CI, Graham CM, Berry MP, Wilkinson KA, Oni T, Rozakeas F, Xu Z, Rossello-Urgell J, Chaussabel D, Banchereau J (2012). Detectable changes in the blood transcriptome are present after two weeks of antituberculosis therapy. PLoS One.

[60] Mayer-Barber KD, Sher A (2015). Cytokine and lipid mediator networks in tuberculosis. Immunol Rev.

[61] Rothchild AC, Stowell B, Goyal G, Nunes-Alves C, Yang Q, Papavinasasundaram K, Sassetti CM, Dranoff G, Chen X, Lee J (2017). Role of granulocyte-macrophage Colony-stimulating factor production by T cells during Mycobacterium tuberculosis infection. MBio.

[62] Becher B, Tugues S, Greter M (2016). GM-CSF: from growth factor to central mediator of tissue inflammation. Immunity.

[63] Baird TD, Wek RC (2012). Eukaryotic initiation factor 2 phosphorylation and translational control in metabolism. Adv Nutr.

[64] Wek RC, Jiang HY, Anthony TG (2006). Coping with stress: eIF2 kinases and translational control. Biochem Soc Trans.

[65] Takigawa S, Chen A, Nishimura A, Liu S, Li BY, Sudo A, Yokota H, Hamamura K (2016). Guanabenz downregulates inflammatory responses via eIF2alpha dependent and independent signaling. Int J Mol Sci.

[66] Redelman-Sidi G, Michielin O, Cervera C, Ribi C, Aguado JM, Fernandez-Ruiz M, Manuel O. ESCMID study Group for Infections in compromised hosts (ESGICH) consensus document on the safety of targeted and biological therapies: an infectious diseases perspective-immune checkpoint inhibitors, cell adhesion inhibitors, sphingosine 1-phosphate receptor modulators and proteasome inhibitors. Clin Microbiol Infect. 2018;(18)30147-2. 10.1016/j.cmi.2018.01.029. 10.1016/j.cmi.2018.01.030 PMC5971148 29427804

[67] Guo Y, Luan L, Patil NK, Sherwood ER (2017). Immunobiology of the IL-15/IL-15Ralpha complex as an antitumor and antiviral agent. Cytokine Growth Factor Rev.

