# Supplementary figures and images for: “Tuberculosis in advanced HIV infection is associated with increased expression of IFNγ and its downstream targets”
Source: BMC Infect Dis. 2018 May 15;18:220. doi: 10.1186/s12879-018-3127-4 (PMC5952419; doi:10.1186/s12879-018-3127-4)

## Slide 1
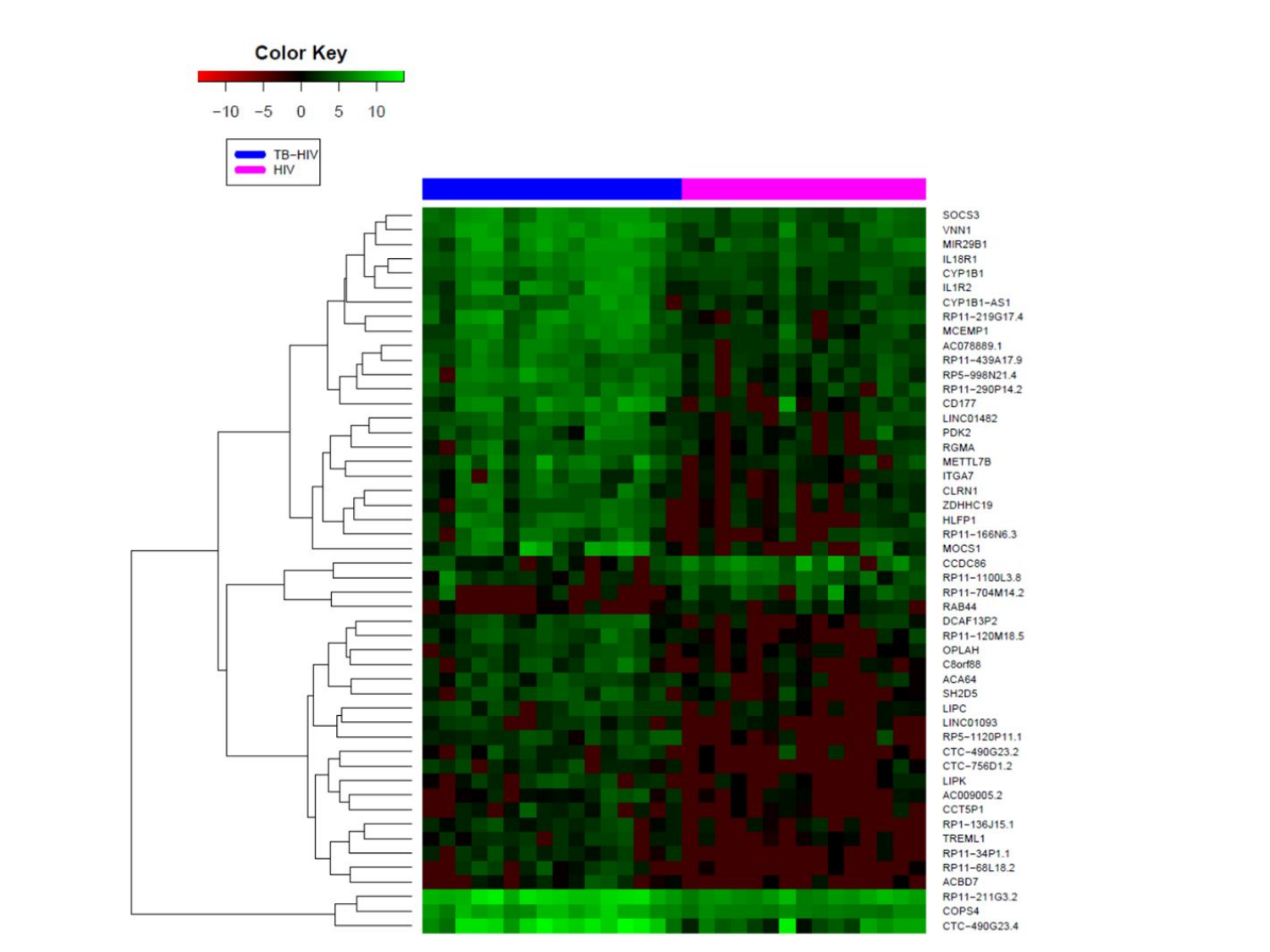

Supplement: Supplementary file 1 — Figure S1. A heatmap of top 50 genes differentially expressed between TB-HIV and HIV. Heat map showing differential gene expression of top 50 genes using largest value of adjusted fold change and p = 0.01 in TB-HIV as compared to HIV-only group. The x-axis is log2 of the fold change between the two groups. The y-axis is log odds of the FDR adjusted p value. (PPTX 297 kb) [file 12879_2018_3127_MOESM1_ESM.pptx]

## Slide 1
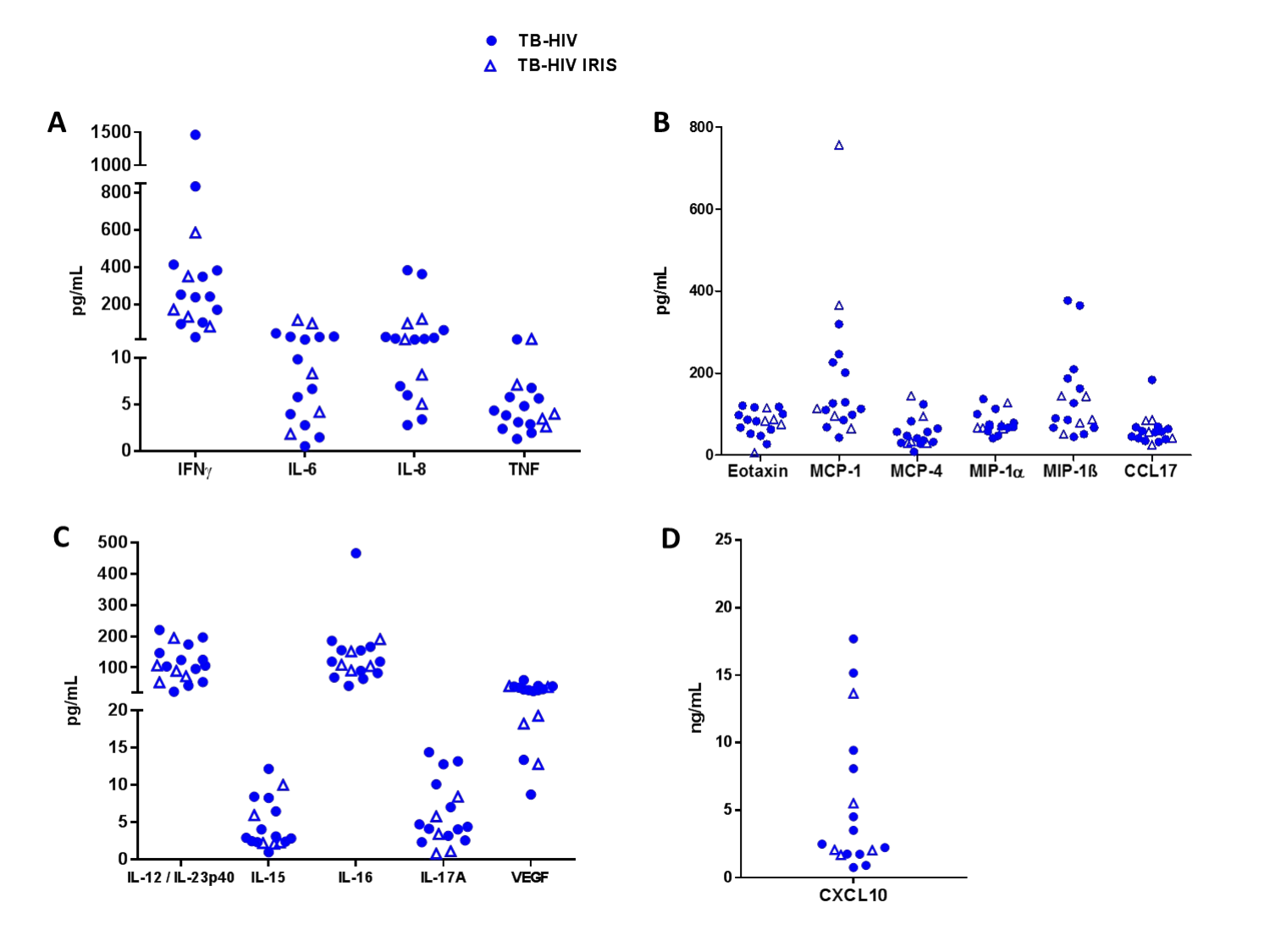

Supplement: Supplementary file 2 — Figure S2. Progression to IRIS does not alter plasma cytokine/chemokine levels in TB-HIV participants. Plasma samples obtained from TB-HIV and TB-HIV with IRIS were analyzed for cytokines and chemokines using the Meso Scale Discovery 30-plex multi-analyte detection system. Data is represented as absolute value for each participant for a given cytokine or chemokine. Significance was determined by Mann Whitney U test. (PPTX 121 kb) [file 12879_2018_3127_MOESM2_ESM.pptx]
